# In vitro toxicological evaluation of aerosols generated by a 4th generation vaping device using nicotine salts in an air-liquid interface system

**DOI:** 10.1186/s12931-024-02697-2

**Published:** 2024-02-05

**Authors:** Clément Mercier, Jérémie Pourchez, Lara Leclerc, Valérie Forest

**Affiliations:** Mines Saint-Etienne, Université Jean Monnet, INSERM, U1059 Sainbiose, Centre CIS, Saint-Etienne, 42023 France

**Keywords:** Electronic cigarette, Toxicology, Aerosol, Air-liquid interface, 4th generation device, Nicotine salts

## Abstract

**Background:**

Electronic cigarettes (EC) have gained popularity, especially among young people, with the introduction of fourth-generation devices based on e-liquids containing nicotine salts that promise a smoother vaping experience than freebase nicotine. However, the toxicological effects of nicotine salts are still largely unknown, and the chemical diversity of e-liquids limits the comparison between different studies to determine the contribution of each compound to the cytotoxicity of EC aerosols. Therefore, the aim of this study was to evaluate the toxicological profile of controlled composition e-liquid aerosols to accurately determine the effects of each ingredient based on exposure at the air-liquid interface.

**Methods:**

Human lung epithelial cells (A549) were exposed to undiluted aerosols of controlled composition e-liquids containing various ratios of propylene glycol (PG)/vegetable glycerin (VG) solvents, freebase nicotine, organic acids, nicotine salts, and flavoured commercial e-liquids. Exposure of 20 puffs was performed at the air-liquid interface following a standard vaping regimen. Toxicological outcomes, including cytotoxicity, inflammation, and oxidative stress, were assessed 24 h after exposure.

**Results:**

PG/VG aerosols elicited a strong cytotoxic response characterised by a 50% decrease in cell viability and a 200% increase in lactate dehydrogenase (LDH) production, but had no effects on inflammation and oxidative stress. These effects occurred only at a ratio of 70/30 PG/VG, suggesting that PG is the major contributor to aerosol cytotoxicity. Both freebase nicotine and organic acids had no greater effect on cell viability and LDH release than at a 70/30 PG/VG ratio, but significantly increased inflammation and oxidative stress. Interestingly, the protonated form of nicotine in salt showed a stronger proinflammatory effect than the freebase nicotine form, while benzoic acid-based nicotine salts also induced significant oxidative stress. Flavoured commercial e-liquids was found to be cytotoxic at a threshold dose of ≈ 330 µg/cm².

**Conclusion:**

Our results showed that aerosols of e-liquids consisting only of PG/VG solvents can cause severe cytotoxicity depending on the concentration of PG, while nicotine salts elicit a stronger pro-inflammatory response than freebase nicotine. Overall, aerosols from fourth-generation devices can cause different toxicological effects, the nature of which depends on the chemical composition of the e-liquid.

## Background

Since their introduction to the market in 2005 (Europe) and 2007 (United States), electronic cigarettes (EC) have quickly gained popularity [1]. Even if vaping continues to generate controversial health and scientific debate, EC are considered by some health authorities to be an effective smoking cessation tool [2, 3]. In addition, EC are generally considered as a less harmful alternative to smoking [4]. ENDS are essentially battery-powered devices that deliver an aerosol to the user by heating a solution known as an e-liquid, resulting in a change of state from liquid to gas [5]. Although the components and operating procedures are similar, the technology of EC has evolved greatly over the years, from the first “cig-a-like” generation to the fourth generation known as “Pod-Mod” devices. Pod-Mods are a class of vaping products with a pre-filled or refillable ‘‘pod’’ or pod cartridge with a modifiable “mod” system (“Pod-Mods”) [6]. Pod-Mods are thus compact and easy-to-use “all-in-one” low-power products EC [7]. . Thanks to these features combined with an appealing, discreet design, this latest generation of EC has gained tremendous popularity (especially among young people), mainly due to the launch of the famous JUUL brand in the US in 2015. Since then, it was estimated that JUUL accounted for about 70% of the sales of EC in 2018, especially among teenagers and young adults [8]. Interestingly, the success of JUUL devices failed to repeat in Europe, resulting in the product being withdrawn from the market in several countries, partly due to the 20 mg/mL maximum nicotine concentration cap enforced by European regulations [9]. Nevertheless, the use of JUUL devices and other Pod-Mods continued to increase in England from 2016 to 2020 [10]. Recently, a modified European branded version of the JUUL device was released to generate a larger aerosol volume and delivers a similar amount of nicotine per puff as US Pod-Mods with a nicotine concentration of 60 mg/mL [11, 12], which may further stimulate JUUL sales in Europe.

, The success of the JUUL and other Pod-Mods brands around the world also seems to be due to the systematic use of nicotine salt-based e-liquids adapted to this type of vaping device. Nicotine salt-based e-liquids are made by adding a weak organic acid that lowers the pH and induces the protonation of nicotine freebase [13]. The protonated form of nicotine appears to the vaper to be more pleasant to taste and much less difficult to inhale than the freebase nicotine at similar nicotine concentrations [14]. This sensory property is one of the main features of inhaling aerosols from nicotine salt-based e-liquids, allowing the vaper to use a higher concentration of protonated nicotine (up to 60 mg/mL in the US market). In addition, several studies reported that protonated nicotine passes the alveolar-capillary barrier more rapidly and to a greater extent to enter the systemic circulation than freebase nicotine for the same initial concentration of nicotine in the e-liquid [15, 16]. While high nicotine levels can facilitate smoking cessation by quickly relieving cravings, they also likely increase the likelihood of long-term dependence in young users with an eventual transition to traditional tobacco products such as conventional cigarettes [17]. In addition to JUUL devices, the ability to deliver high doses of nicotine with little discomfort has led to the introduction of similar Pod-Mod devices from various brands and the proliferation of thousand nicotine salt-based e-liquids in the market [18].

The commercial development of e-liquids containing nicotine salts has further complicated the chemical diversity of the composition of e-liquids available on the market. Indeed, the formulation of e-liquids includes several categories of ingredients. Basically, the humectants propylene glycol (PG) and vegetable glycerin (VG) are used in almost all e-liquids, which may also contain nicotine (in its freebase or protonated form). The composition of e-liquids also includes a variety of flavours [19]. Although humectants (PG and VG) and many flavourings are “generally regarded as safe” (GRAS) by the Food and Drug Administration (FDA) in food or pharmaceutical products [20, 21], the conclusions have mostly been drawn from oral intake studies in animals, whereas the pulmonary toxicity of GRAS has hardly been studied. However, some studies suggested that concentrations of PG and VG in EC puffs may be sufficient to cause irritation or injury to the respiratory tract [22, 23]. Conversely, pulmonary and cardiovascular cytotoxicity of several flavouring compounds has been extensively studied and demonstrated [24, 25]. In addition to the intrinsic toxicity of e-liquid ingredients, the heating process can also lead to the generation of new thermal decomposition compounds that could also have harmful effects. Indeed, approximately 250 chemicals have been identified in aerosols from EC, including both the original ingredients of e-liquids (PG/VG, nicotine, and flavourings) and numerous potentially harmful thermal degradations products, the most abundant being carbonyl compounds and volatile organic compounds (VOC) [26, 27]. Although Pod-Mods devices operate at much lower temperatures than 3rd generation devices EC, these devices have been shown to produce trace amounts of thermal degradation products [28, 29]. In addition, several toxic metals have been detected in both e-liquids and aerosols from POD-based devices, the concentrations of which may increase over time due to repeated heating of the coil by refilling, a practice not recommended by the manufacturer [30, 31].

To date, few studies have highlighted the toxicity of aerosols produced by 4th generation vaping devices and the specific effects of nicotine protonation levels [32, 33]. However, it is important to note that these studies were performed in vitro under submerged culture condition and aerosol exposure, which do not mimic physiological exposure of the respiratory tract. Interestingly, recent studies have provided new evidence for the toxicity of Pod-Mods emissions, showing that EC aerosols of nicotine salts induce profound changes in oxidative stress-related genes in macrophages and alter cytokine expression patterns in airway epithelial cells [34, 35].

Although more studies have been conducted recently on the pulmonary toxicity of 4th generation EC aerosols using nicotine salt-based e-liquids representative of the US market (60 mg/mL nicotine), there is still a lack of knowledge on the specific toxicity of aerosols produced by 4th generation vaping devices using nicotine salts at concentrations representative of the European market (i.e., 20 mg/mL or maximum 2% nicotine). More importantly, the diversity and chemical complexity of available e-liquids requires the development of well-designed in vitro studies to accurately control aerosol generation and chemical composition to demonstrate the individual impact of each e-liquid ingredients. Therefore, the present study was designed to investigate the toxicological profile of each e-liquid ingredients after vaporization on lung epithelial cells using an air-liquid interface exposure system. For this purpose, epithelial cells were exposed to undiluted aerosols of PG/VG humectants, freebase nicotine, organic acids, nicotine salts, and flavoured commercial e-liquids at the physiological air-liquid interface following a standardized puffing regimen in a validated exposure chamber. We then evaluated the toxicological profile of the aerosols by assessing cell viability, membrane integrity, induction of inflammation, and oxidative stress.

## Materials and methods

### Chemicals and reagents

Propylene glycol (PG), vegetable glycerin (VG), (-)-nicotine, benzoic acid and salicylic acid were purchased from Sigma-Aldrich. Purity was > 99% for each compound. JUUL Mint (30/70 PG/VG ratio and 18 mg/mL nicotine) and Flavor Power Mint (50/50 PG/VG ratio and 18 mg/mL nicotine) e-liquids were purchased from JUUL online store and “Le vapoteur discount” online store, respectively. Cell culture reagents were obtained from Gibco (Waltham, MA).

### Cell culture

The A549 lung epithelial cell line was obtained from the American Type Culture Collection (ATCC - CCL-185) and routinely maintained in Dulbecco’s Minimum Essential Medium (DMEM) supplemented with 10% fetal bovine serum (FBS), 1% L-glutamine, and 1% antibiotics/antimycotics (penicillin-streptomycin, amphotericin B solution). Cells were incubated at 37 °C and 5% CO_2_ and used between passages P10 and P20. For aerosol exposure at the air-liquid interface (ALI), A549 cells were seeded at a density of 6*10^3^ cells/cm² on porous ThinCert polyethylene terephthalate (PET) culture inserts with a growth area of 0.33 cm² and a pore size of 0.4 μm (Greiner, 662,641). Cells were grown under submerged conditions, with culture medium filled in both compartments and renewed every two days for four days. The next day, the apical medium was removed, and the culture was raised to ALI in 350 µL of basolateral medium. Cells were allowed to differentiate in ALI for 24 h before exposure to aerosol.

### E-liquids preparation

**Fig. 1:**
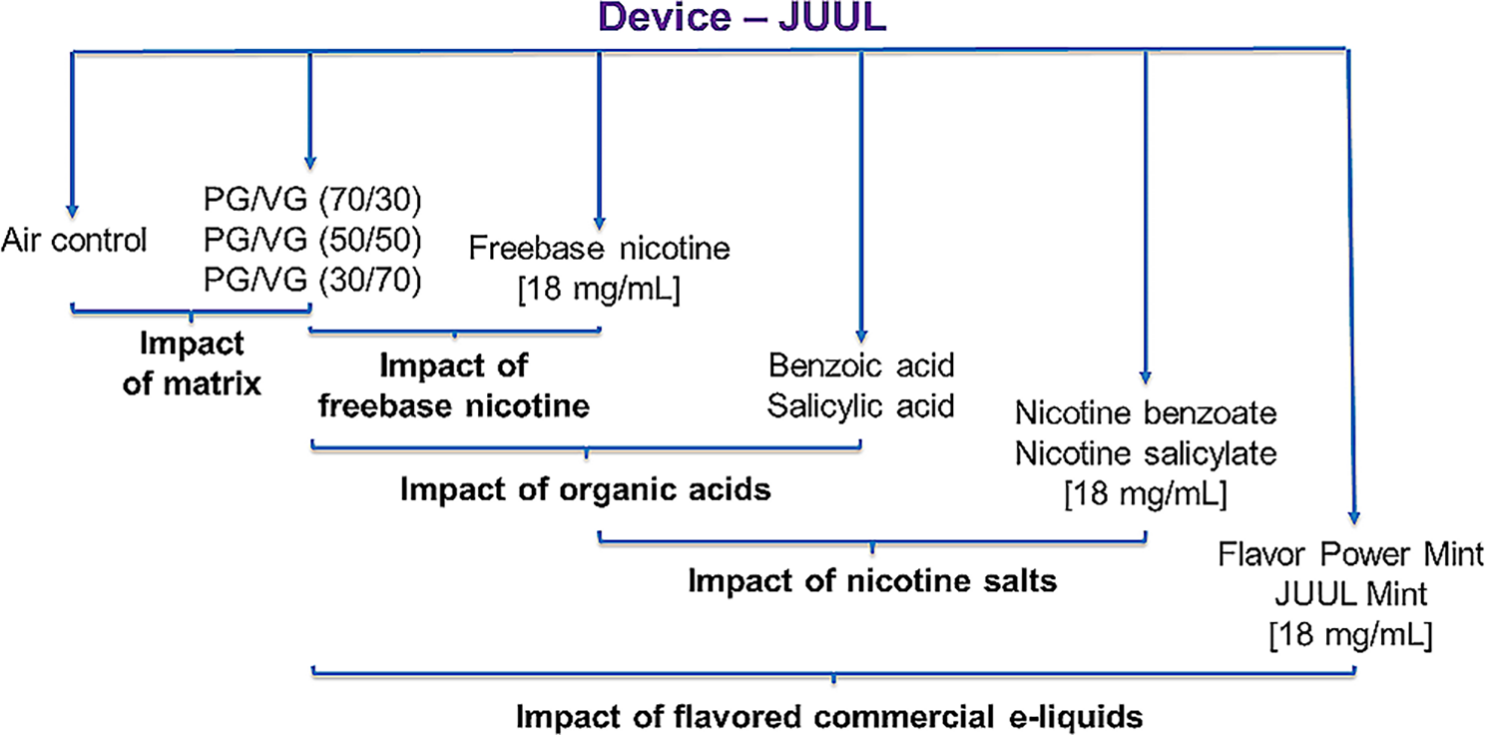
Experimental conditions and relevant relative comparisons

Ten e-liquids were analysed sequentially to cover a wide range of e-liquid compositions and to evaluate the specific toxicological impact of each ingredientinvolved in the formulation (Fig. 1; Table 1). Three PG/VG ratios were prepared (30/70, 50/50, and 70/30), with only the 70/30 ratio used as the base matrix for the preparation of all e-liquids except commercials. E-liquids were prepared by diluting nicotine alone (freebase nicotine), organic acids alone, or nicotine salts (mixture of freebase nicotine and organic acids) in a ratio of 70/30 PG /VG. A 1:1 molar ratio of nicotine:acid was used to prepare nicotine salts. Commercially available e-liquids were purchased directly from online stores. JUUL Pods were dismounted and emptied before being thoroughly washed in a 50:50 (v/v) ethanol:water solution in ultrasonic bath during 20 min. This operation was repeated three times and Pods were dried at 60 °C during 2 h. The clean Pods were then filled with the prepared or commercial e-liquids.

**Table 1:** Experimental conditions and e-liquids composition

	Propylene glycol	Vegetable glycerin	Benzoic acid	Salicylic acid	Nicotine	Flavours
**Control**						
Air	-	-	-	-	-	-
**Matrix**						
PG/VG 70/30 (v/v)	70%	30%	-	-	-	-
PG/VG 50/50 (v/v)	50%	50%	-	-	-	-
PG/VG 30/70 (v/v)	30%	70%	-	-	-	-
**Nicotine freebase**	70%	30%	-	-	18 mg/mL	-
**Organic acids**						
Benzoic acid	70%	30%	13.5 mg/mL	-	-	-
Salicylic acid	70%	30%	-	15.3 mg/mL	-	-
**Nicotine salts** molar ratio nicotine:acid 1:1						
Nicotine benzoate	70%	30%	13.5 mg/mL	-	18 mg/mL	-
Nicotine salicylate	70%	30%	-	15.3 mg/mL	18 mg/mL	-
**Commercial e-liquids**						
Flavor Power Mint	50%	50%	-	+*	18 mg/mL	Mint
JUUL Mint	30%	70%	+*	-	18 mg/mL	Mint

* Concentration not disclosed by the manufacturer

### Validation of exposure setup

**Fig. 2:**
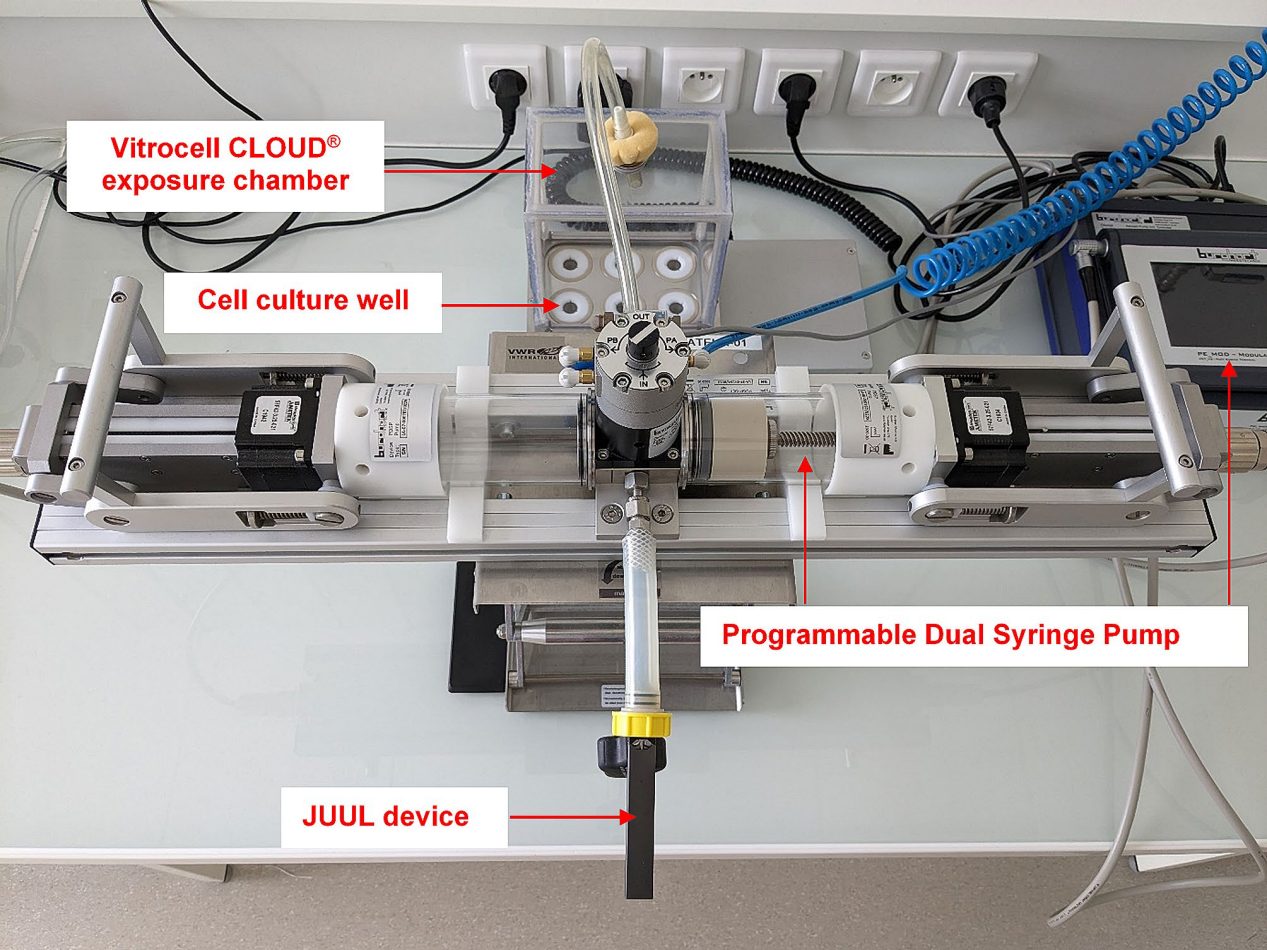
Overview of the exposure setup

The exposure setup was evaluated in terms of average deposited dose and uniformity of dose deposition in all culture wells. As shown in Fig. 2, the JUUL device was connected to a modular puffing machine based on an automated and programmable Dual Syringe Pump (Burghart, Germany), which in turn was connected to the 6-culture wells Vitrocell CLOUD (Vitrocell Systems GMBH, Waldkirch, Germany). To determine the average dose deposition, three 44-milimeter glass filters were weighed and placed in the chamber before exposure. 20 puffs of each e-liquid were then aerosolized according to AFNOR standard XP D90-300-3: 3 s puff, 55 mL/puff, 30 s between puffs [36], and aerosols were allowed to settle on the filters for 20 min before being reweighed. All exposures were performed in triplicate. To assess uniformity of deposition, six filters were weighed and spread over each culture well in the chamber before being exposed to 20 puffs of 70/30 PG/VG aerosol. After 20 min of incubation, the filters were reweighed to determine the final mass. Data represent the mean of five independent experiments. The deposited dose was calculated for each dosimetry experiment using the following Eq. 1:

$$\begin{array}{l}Deposited{\mkern 1mu} {\mkern 1mu} dose\left( {\mu g{\rm{/c}}{{\rm{m}}^{\rm{2}}}} \right) = \\\,\,\,\,\,\,\,\,\,\,\,\,\,\frac{{Final{\mkern 1mu} filter{\mkern 1mu} {\mkern 1mu} mass\left( {\mu g} \right) - Initial{\mkern 1mu} filter{\mkern 1mu}{\mkern 1mu} mass\left( {\mu g} \right)}}{{Filter{\mkern 1mu} surface\left( {c{m^2}} \right)}}\end{array}$$

### Aerosol generation and cell exposures

**Fig. 3:**
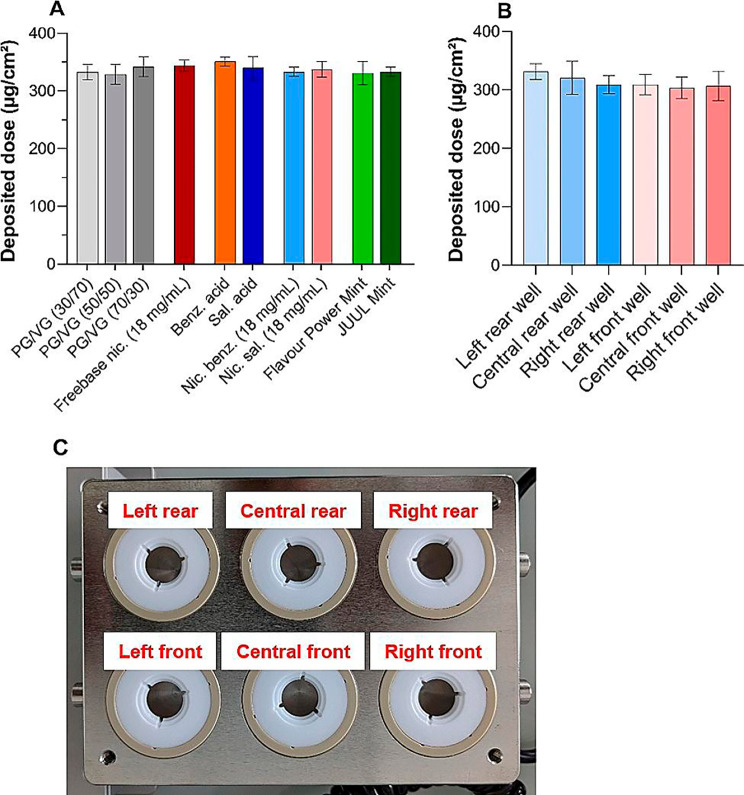
Validation of Vitrocell CLOUD exposure chamber. (**A**) 20 puffs of aerosol of each e-liquid were aerosolised in the chamber and the deposited dose was assessed by a gravimetric method. (**B**) 20 puffs of 70/30 PG/VG aerosol were aerosolized in the exposure chamber and deposited dose was calculated according to (**C**) the cell culture well position in the chamber Results are presented as mean ± SD for *n* = 3 (**A**) and *n* = 5 (**B**) independent experiments

Aerosol generation was performed using a 4th generation JUUL device (JUUL Labs, US). The exposure setup consisted of an aerosol generation module coupled to an exposure chamber (Fig. 2). To generate e-cigarette aerosols, the JUUL device was connected to a modular puff engine based on an automated and programmable Dual Syringe Pump (Burghart, Germany). The syringe outlet port was connected to the 6-culture wells of the Vitrocell CLOUD (Vitrocell Systems GMBH, Waldkirch, Germany) exposure chamber equipped with a built-in temperature controller set at 37 °C. 24 h post- ALI, the inserts were quickly transferred to the exposure chamber and secured in the wells filled with 3.2 mL of prewarmed complete culture medium on the basolateral side to maintain the cells during the experiments. A 44-millimeter glass filter was weighed and placed on the right front well in the chamber for dosimetry assessment (Fig. 3C). Cells were exposed to 20 puffs of undiluted e-cigarette aerosols drawn according to AFNOR standard XP D90-300-3 (AFNOR), and aerosols were allowed to settle on the culture inserts for 20 min. After exposure, the inserts were quickly removed from the chamber, returned to the 24-well plates, and then incubated at 37 °C for 24 h before the basolateral media were collected and stored at -80 °C for toxicological assays. The glass filter was then reweighted to calculate the deposited dose according to Eq. 1. Each batch of inserts was exposed to medical air as an internal negative control. The entire exposure setup was thoroughly cleaned with 70% ethanol and distilled water between each exposure series to avoid cross-contamination. Three independent exposures were performed in triplicate for each e-liquid (total *n* = 9).

### Cell viability

Cell viability was evaluated 24 h after aerosol exposure using CellTiter-Blue® assay. The assay is based on the ability of viable cells to convert resazurin dye into fluorescent product resofurin, thus reflecting cell metabolic activity. Briefly, inserts were retrieved and incubated at 37 °C for 2 h with 20 µL of reagent diluted in 100 µL of complete culture medium filled into the apical compartment. After incubation, supernatants were transferred to a 96-well plate and fluorescence was read using a fluorometer (Fluoroskan Ascent, ThermoFisher Scientific, Waltham, MA) with wavelengths set at 530 nm (excitation) and 590 nm (emission). Fluorescence values for the negative control group of air-exposed cells were set to 1. Results were expressed as a ratio to the negative control.

### Lactate dehydrogenase (LDH) assay

Extracellular release of LDH was quantified in the basolateral culture media 24 h post-exposure to aerosol using the CytoTox-96® Non-Radioactive Cytotoxicity Assay (Promega, Madison, WI, USA) according to the manufacturer’s instructions. Optical density was determined using a microplate reader at 490 nm (Multiskan GO, ThermoFisher Scientific, Waltham, MA, USA) and released LDH was expressed as a percentage of the maximum LDH released from lysed cells (positive control).

### Interleukin-8 (IL-8) production

The production of the pro-inflammatory cytokine interleukin-8 was assessed using the human IL-8 ELISA kit (ThermoFisher Scientific, Waltham, MA, USA) according to the manufacturer’s instructions. Samples were read with a microplate reader (Multiskan GO) set to 450 nm. IL-8 production was quantified from a standard curve and expressed as a ratio to the negative control of air-exposed cells set to 1.

### 8-hydroxy 2 deoxyguanosine (8-OHdG) production

The 8-hydroxy 2 deoxyguanosine ELISA Kit (Abcam, Cambridge, United Kingdom) was used to quantify the production of 8-OHdG as a marker of oxidative stress in the basolateral culture media 24 h post-exposure to aerosol. The assay was performed according to the manufacturer’s instructions. The optical density of each sample was measured with a microplate reader (Multiskan GO) at 450 nm. 8-OHdG production was quantified from a standard curve and expressed as a ratio to the negative control of air-exposed cells set to 1.

### Statistical analysis

Results of each biological assay are expressed as mean ± standard deviation (SD) of 3 independent experiments, each performed in triplicate. Data were processed using GraphPad Prism 9 software (GraphPad Software, San Diego, CA). Two-sided Student’s t-test was used for pairwise comparisons. Kruskal-Wallis test followed by a Dunn’s post-hoc test was used when testing differences between 3 or more groups. *p*-value < 0.05 was considered statistically significant.

## Results

### Validation of aerosol exposure reproducibility at the air-liquid interface

Characterization of the exposure chamber with experimental assessment of the aerosol dose deposited on the cells is a crucial point to be considered for robust comparative toxicological studies of EC emission. Precisely, the ability of the exposure system to deliver equal aerosol doses between each condition is a fundamental requirement. Our results showed that the deposited dose ranged from 328.8 ± 13.2 µg/cm² (for the “50/50 PG /VG” condition) to 350.7 ± 6.6 µg/cm² (for the “benzoic acid” condition), with no significant statistical differences (*p* = 0.70) among the studied e-liquids (Fig. 3A). The average deposited dose for the 10 experimental conditions was 339.5 ± 19.8 µg/cm², indicating low variability in aerosol mass deposition on cell wells. Furthermore, the generation of 20 puffs of 70/30 PG/VG resulted in uniform aerosol deposition within the exposure chamber and no significant statistical differences (*p* = 0.24) were observed between each cell culture wells (Fig. 3B and C). Taken together, these data underline the reliability and validate the EC exposure system for further toxicological studies of aerosols generated using a 4th generation device.

### PG level in the e-liquid is the main driver of cytotoxicity

**Fig. 4:**
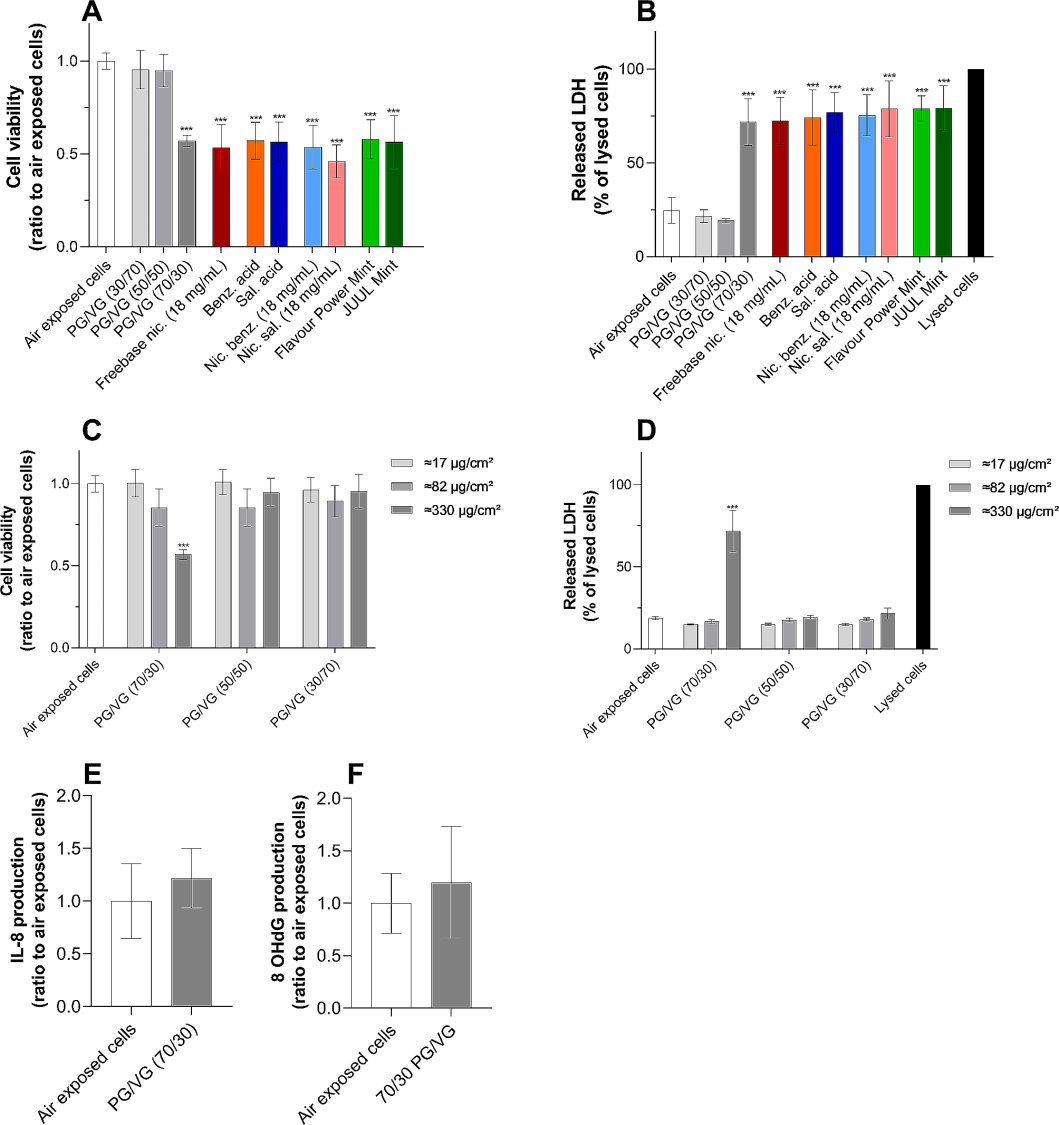
70/30 PG/VG is the main driver of cytotoxicity but has no impact on inflammation and oxidative stress. A549 cells were exposed to ≈ 330 µg/cm² of each e-liquid aerosol and toxicological endpoints were assessed 24 h post-exposure. All e-liquids but Flavour Power Mint and JUUL Mint were based on a 70/30 PG/VG ratio. (**A**) Cell viability was evaluated with CellTiter-Blue® (rezasurin). Data are expressed as a ratio to air-exposed cells. (**B**) Released LDH in the supernatant of A549 cells. Data are expressed as a percentage of maximum LDH release in lysed cells. (**C, D**) A549 cells were exposed to ≈ 330 µg/cm² aerosols of three PG/VG ratios (30/70, 50/50 and 70/30) at three doses (≈ 17 µg/cm², ≈ 82 µg/cm² and ≈ 330 µg/cm²), and (**C**) cell viability and (**D**) released LDH were evaluated 24 h post-exposure. (**E**) IL-8 and (**F**) 8-OHdG production in A549 cells 24 h post-exposure to ≈ 330 µg/cm² of a 70/30 PG/VG aerosol. Data are expressed as a ratio to air-exposed cells. All results are presented as mean ± SD from 3 independent experiments, each with 3 technical replicates per condition. ***p ≤ 0.001 as compared to air exposed cells as determined by Kruskal-Wallis test followed by Dunn’s post-hoc test. Freebase nic.: freebase nicotine, benz. acid: benzoic acid, sal. acid: salicylic acid, nic. benz.: nicotine benzoate, nic. sal.: nicotine salicylate

A549 cell model was exposed to ≈ 330 µg/cm² aerosols of 10 e-liquids of varying composition and increasing chemical complexity. All 10 experimental conditions, except 50/50 and 30/70 PG/VG, significantly decreased cell viability by approximately 50% compared with cells exposed to air (Fig. 4A). The decrease in cell viability was accompanied by an almost threefold increase in LDH release in aerosol-exposed cells compared with air-exposed control cells, suggesting alterations in membrane integrity (Fig. 4B). Surprisingly, aerosol of only 70/30 PG/VG exerted strong cytotoxicity in both assays. Because the 70/30 PG/VG ratio was used as the common base matrix for all reference e-liquids, the PG/VG solvents constituted the main source of cytotoxicity. Interestingly, both the 50/50 and 30/70 ratios did not elicit LDH release or loss of viability regardless of dose, suggesting that the concentration of PG may be primarily responsible for cytotoxicity (Fig. 4C and D). In addition, the toxicity of the PG /VG matrix was dose-dependent, with no effects observed at doses of 1 (≈ 17 µg/cm²) and 5 puffs (≈ 82 µg/cm²). Moreover, 70/30 PG/VG aerosol only slightly increased IL-8 and 8-OHdG production compared with cells exposed to air-exposed cells suggesting no effects on inflammatory and oxidative stress processes (Fig. 4E and F). Overall, our results showed that aerosols of PG/VG solvents could trigger cytotoxic effects above a threshold dose of about 330 µg/cm². Since these effects occurred only at a ratio of 70/30, the PG level in the e-liquid can be considered as the main driver of aerosol cytotoxicity.

### Freebase nicotine is the main driver of the oxidative stress

**Fig. 5:**
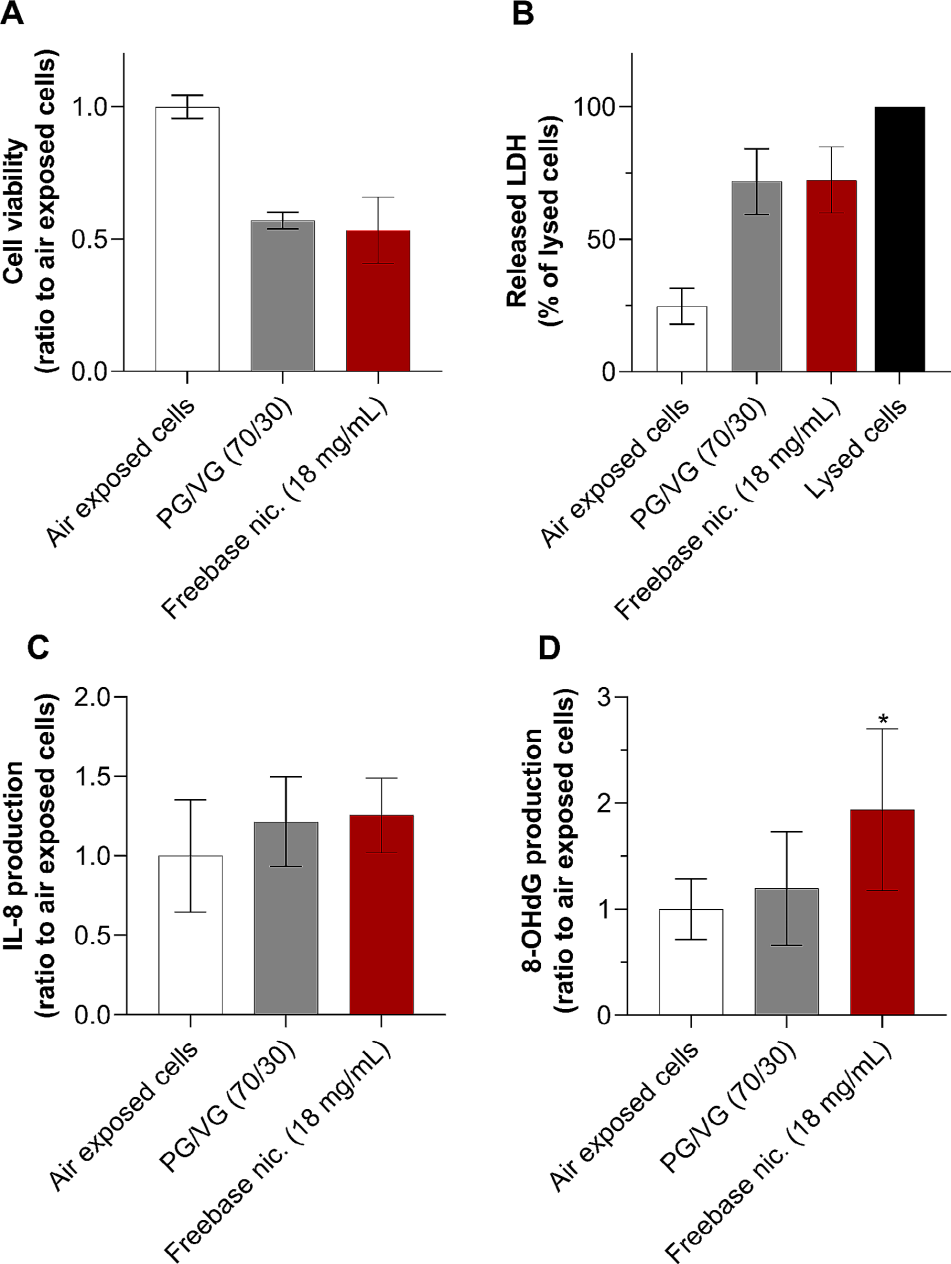
Freebase nicotine induces oxidative stress. A549 cells were exposed to pure air control, and ≈ 330 µg/cm² aerosols of 70/30 PG/VG and freebase nicotine at 18 mg/mL in 70/30 PG/VG. Toxicological endpoints were assessed 24 h post-exposure. (**A**) Cell viability was evaluated with CellTiter-Blue® (rezasurin). Data are expressed as a ratio to air-exposed cells. (**B**) Released LDH in the supernatant of A549 cells. Data are expressed as a percentage of maximum LDH release in lysed cells. (**C**) IL-8 and (**D**) 8-OHdG production in the supernatant of A549 cells. Data are expressed as a ratio to air-exposed cells. All results are presented as mean ± SD for *n* = 3 independent experiments, each with 3 technical replicates per condition. *p ≤ 0.05 as compared to PG/VG (70/30) as determined by Kruskal-Wallis test followed by Dunn’s post-hoc test. Freebase nic.: freebase nicotine

We focused on the effects of freebase nicotine in relation to the 70/30 PG/VG matrix. Exposure of cells to nicotine freebase aerosol resulted in only a slight 7% decrease in cell viability, whereas LDH levels remained unchanged compared with the 70/30 PG/VG aerosol (Fig. 5A and B). The production of IL-8 production was also slightly increased (≈ 3%), indicating that freebase nicotine did not elicit any proinflammatory effects beyond those of the PG/VG solvents (Fig. 5C). Conversely, exposure of cells to 20 puffs of freebase nicotine aerosol resulted in a significant 60% increase in 8-OHdG production compared with cells exposed to the 70/30 PG/VG matrix, indicating an intrinsic oxidative potential of freebase nicotine (Fig. 5D). These results suggest that although freebase nicotine freebase aerosols do not induce cytotoxicity and inflammation, they may still be involved in the generation of acute oxidative stress.

### Organic acids are the main drivers of the pro-inflammatory effects

**Fig. 6:**
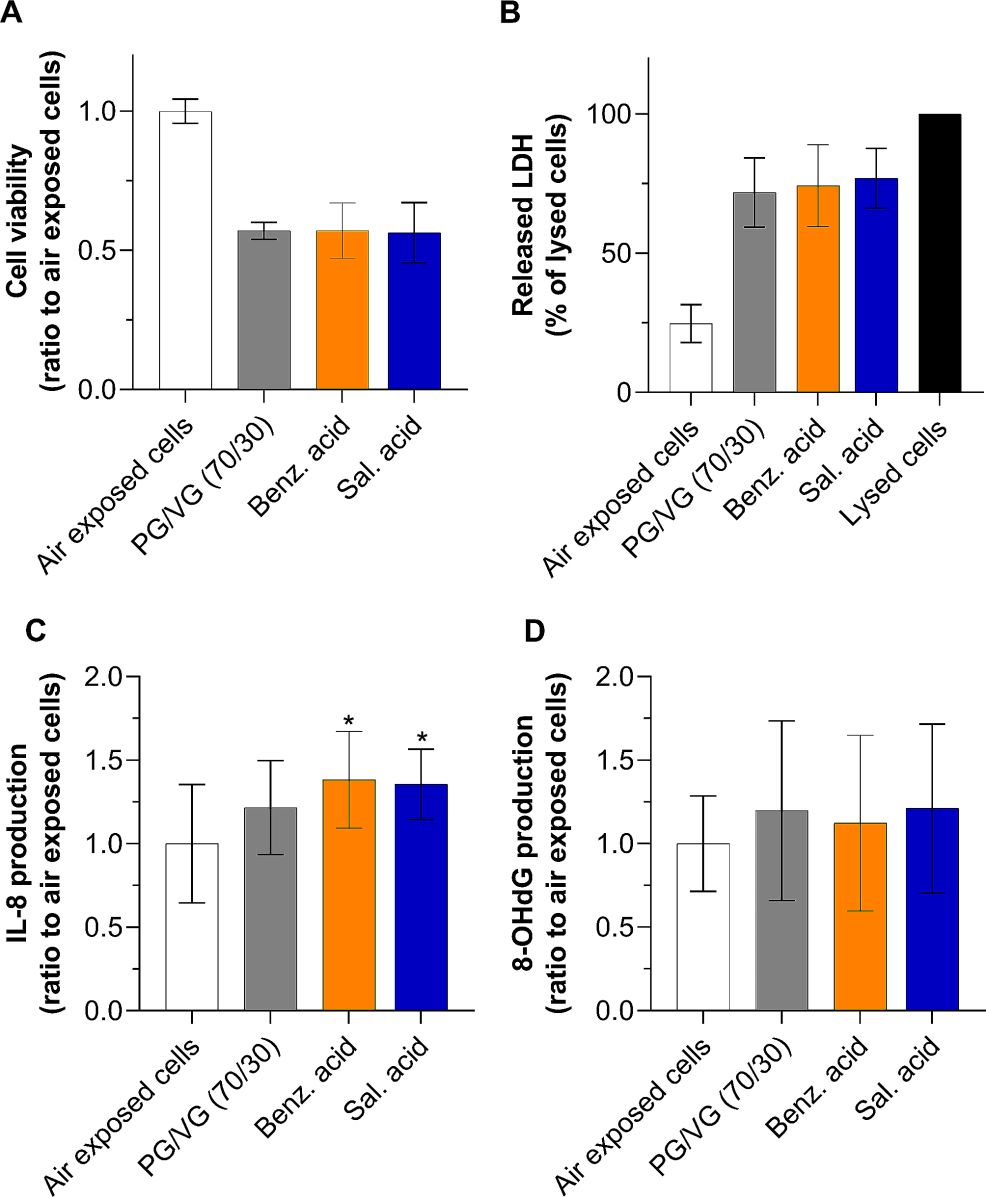
Organic acids in 70/30 PG/VG are pro-inflammatory. A549 cells were exposed to pure air control, and ≈ 330 µg/cm² aerosols of 70/30 PG/VG, benzoic acid and salicylic acid in 70/30 PG/VG. Toxicological endpoints were assessed 24 h post-exposure. (**A**) Cell viability was evaluated with CellTiter-Blue® (rezasurin). Data are expressed as a ratio to air-exposed cells. (**B**) Released LDH in the supernatant of A549 cells. Data are expressed as a percentage of maximum LDH release in lysed cells. (**C**) IL-8 and (**D**) 8-OHdG production in the supernatant of A549 cells. Data are expressed as a ratio to air-exposed cells. All results are presented as mean ± SD for *n* = 3 independent experiments, each with 3 technical replicates per condition. *p ≤ 0.05 as compared to air-exposed cells as determined by Kruskal-Wallis test followed by Dunn’s post-hoc test. Benz.acid: benzoic acid, sal. acid: salicylic acid

Compared with cells exposed to 70/30 PG /VG, exposure to ≈ 330 µg/cm² of benzoic acid and salicylic acid aerosols did not change the amount of LDH released or cell viability (Fig. 6A and B). Also, 8-OHdG production was comparable to that of the 70/30 PG/VG matrix (Fig. 5D). Interestingly, aerosols containing organic acids induced only a small 11% increase in IL-8 production compared with 70/30 PG/VG exposed cells, suggesting that benzoic acid and salicylic acids did not exhibit intrinsic pro-inflammatory properties. However, IL-8 production was significantly higher (≈ 1.36 fold) compared with cells exposed to air alone (Fig. 6C). Taken together, these data provide evidence for a synergistic effect of organic acids on IL-8 production in a 70/30 PG/VG ratio, which may be associated with an inflammatory process.

### Nicotine salts promote both inflammation and oxidative stress

**Fig. 7:**
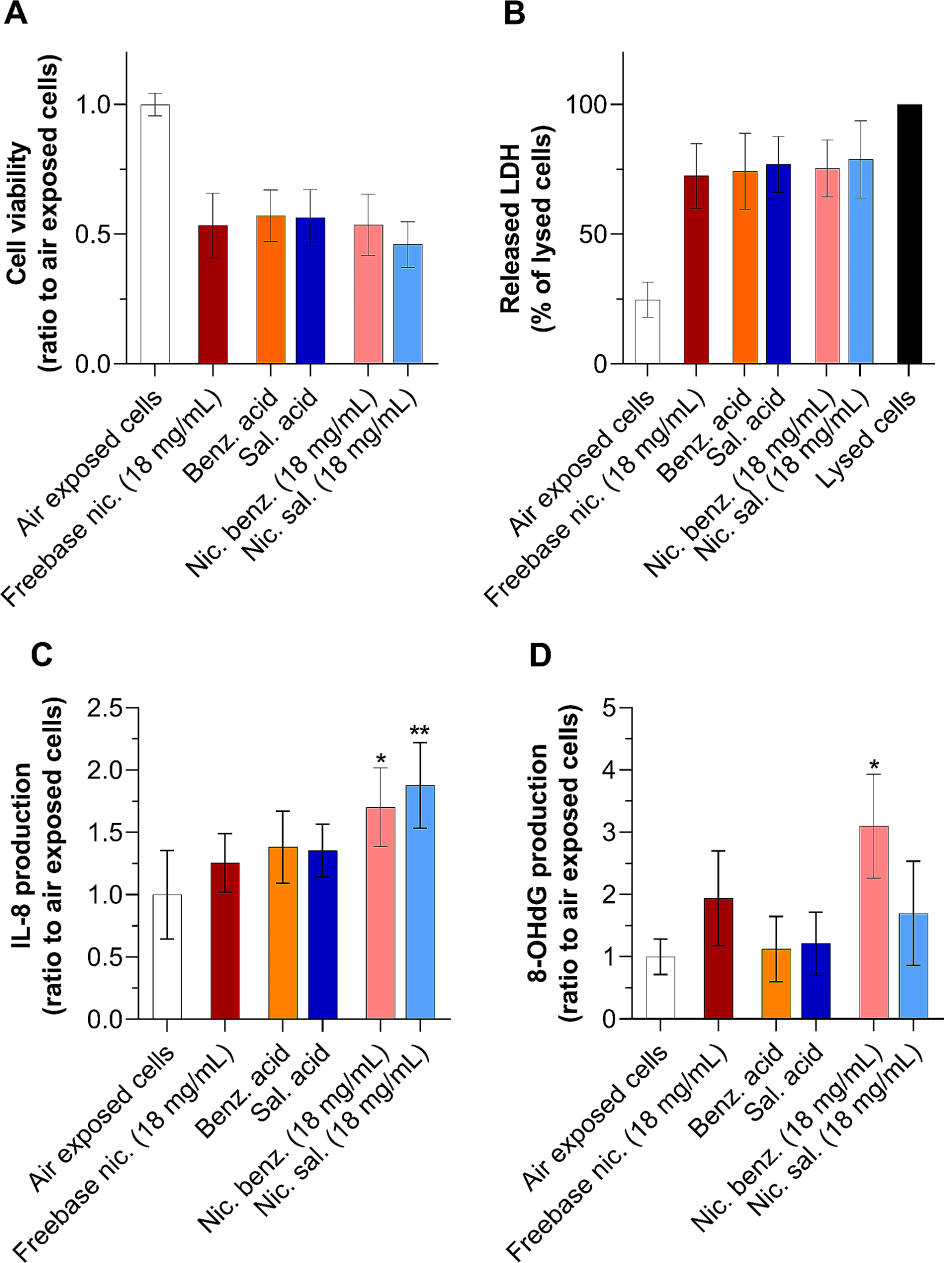
Nicotine salts promote inflammation and oxidative stress. A549 cells were exposed to pure air control, and ≈ 330 µg/cm² aerosols of freebase nicotine (18 mg/mL), benzoic acid, salicylic acid (18 mg/mL), nicotine benzoate (18 mg/mL) and nicotine salicylate in 70/30 PG/VG. Toxicological endpoints were assessed 24 h post-exposure. (**A**) Cell viability was evaluated with CellTiter-Blue® (rezasurin). Data are expressed as a ratio to air-exposed cells. (**B**) Released LDH in the supernatant of A549 cells. Data are expressed as a percentage of maximum LDH release in lysed cells. (**C**) IL-8 and (**D**) 8-OHdG production in the supernatant of A549 cells. Data are expressed as a ratio to air-exposed cells. All results are presented as mean ± SD for *n* = 3 independent experiments, each with 3 technical replicates per condition. *p ≤ 0.05 and **p ≤ 0.01 as compared to freebase nicotine as determined by Kruskal-Wallis test followed by Dunn’s post-hoc test. Benz.acid: benzoic acid, sal. acid: salicylic acid, nic. benz.: nicotine benzoate, nic. sal.: nicotine salicylate

The effect of nicotine salts was evaluated in comparison with freebase nicotine at the same nicotine concentration. Our results showed that cell viability and released LDH levels of cells exposed to nicotine salt aerosols were similar to those of freebase nicotine, indicating only minor cytotoxic effects (Fig. 7A and B). In contrast, nicotine benzoate and nicotine salicylate aerosols strongly increased IL-8 production by approximately 35% and 50%, respectively, compared with freebase nicotine (Fig. 7C). However, no significant differences in the release of IL-8 were observed between both nicotine salts. In addition, cells exposed to nicotine benzoate showed a significant 1.6-fold increase in 8-OHdG production compared with nicotine freebase, whereas 8-OHdG concentrations remained unchanged in cells exposed to nicotine salicylate (Fig. 7D). Finally, our data showed that exposure to nicotine benzoate induced almost twice as high 8-OHdG production as that to nicotine salicylate. Overall, these results showed that nicotine in its salt form is more proinflammatory than its freebase form. In contrast to salicylic acid, the benzoic acid-based nicotine salts also induce significant oxidative stress compared to freebase nicotine.

### Flavoured salt-based commercial e-liquids are cytotoxic

**Fig. 8:**
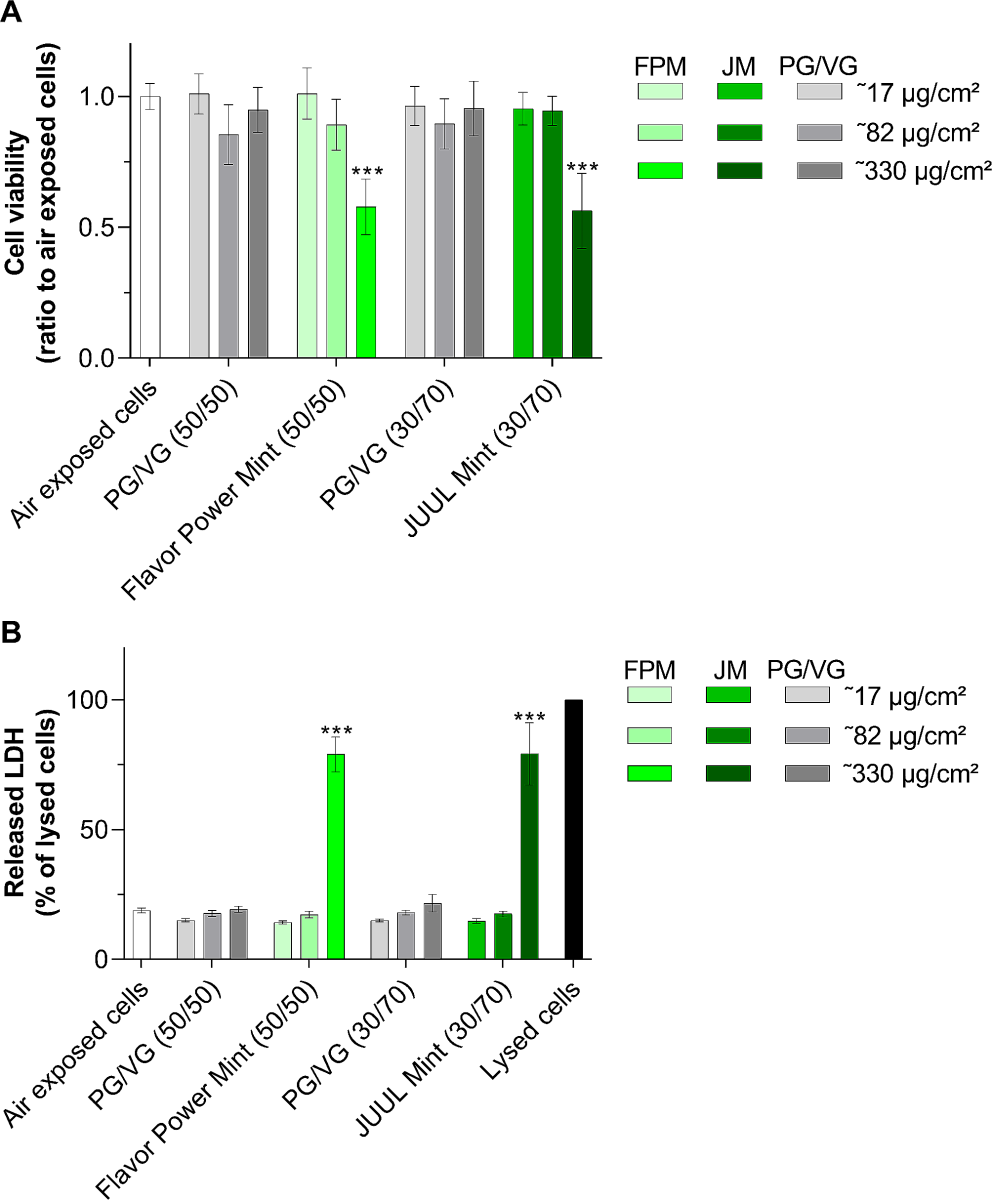
Mint-flavored nicotine salt-based e-liquids are cytotoxic. A549 cells were exposed to to pure air control, and aerosols of Flavor Power Mint and JUUL Mint e-liquids as well as their corresponding 50/50 and 30/70 PG/VG ratios at three doses (≈ 17 µg/cm², ≈ 82 µg/cm² and ≈ 330 µg/cm²). Toxicological endpoints were assessed 24 h post-exposure. (**A**) Cell viability was evaluated with CellTiter-Blue® (rezasurin). Data are expressed as a ratio to air-exposed cells. (**B**) Released LDH in the supernatant of A549 cells. Data are expressed as a percentage of maximum LDH release in lysed cells. All results are presented as mean ± SD for *n* = 3 independent experiments, each with 3 technical replicates per condition. ***p ≤ 0.001 as compared to air exposed cells as determined by Kruskal-Wallis test followed by Dunn’s post-hoc test

Finally, we investigated the toxicological effects of aerosols of commercial e-liquids with complex nicotine salt flavours on the A549 cell model. Because we have shown that a 70/30 PG /VG matrix elicited a strong cytotoxic response, commercial e-liquids with a PG/VG ratio identical to that of their base matrix (50/50 PG /VG and 30/70 PG/VG for Flavor Power Mint and JUUL Mint, respectively) were compared. We found that aerosols of both commercial e-liquids decreased cell viability at the highest dose by approximately 50% compared with their PG /VG base matrix counterparts (Fig. 8A). This was supported by a threefold increase in released LDH, suggesting that commercial e-liquids damage cell membrane integrity (Fig. 8B). Interestingly, cytotoxicity appeared to be dose-dependent, as exposure of cells to lower doses had no effect on cell viability or LDH release. Taken together, these results suggested that the complex chemical composition of commercial nicotine salt-based e-liquids containing PG/VG, nicotine, organic acids, and flavourings could be cytotoxic above a threshold dose.

## Discussion

Over the past decade, the design and technologies of EC have rapidly evolved to better meet consumer habits and cravings for nicotine as well as to support smoking cessation needs. The rising popularity of EC has led to a significant increase in the prevalence of nicotine vaping worldwide, from an estimated 20 million vapers in 2012 to a projected 86 million in 2023 [37]. The recent increase in e-cigarette use among adolescents and young adults is thought to be linked to the introduction of 4th generation Pod-Mod and disposable devices, which combine attractive features such as ease of use, low price, discreet size, and a wide variety of flavours [7]. Despite their exponential popularity among the vulnerable and often misinformed young population, the toxicity of Pod-Mod emissions remains poorly investigated. In addition to the heterogeneity of 4th generation EC devices, the vape market is also flooded with various e-liquids [38, 39]. Although flavours are the main source of diversity in e-liquids, the base formulation ingredients may also vary, contributing to the chemical heterogeneity of e-liquid composition [40]. PG/VG base solvents are available in various ratios, with 30/70, 50/50, and 70/30 being the most popular [41]. The nicotine concentration in European e-liquids can range from 0 to a maximum of 20 mg/mL as required by the European Tobacco Products Directive [8]. Finally, nicotine can also exist in a variety of forms depending on its protonation state driven by pH of the solvent and nicotine pKa [42] and protonated nicotine salts can be generated with a variety of weak organic acids, including benzoic acid, salicylic acid, or lactic acid, among at least six others [43]. We have previously discussed the consequences of the heterogeneity of devices and e-liquids, which leads to a large number of in vitro studies with different experimental designs and aims, ultimately making consistent comparisons and robust conclusions on the toxicity of e-cigarette aerosols difficult [44]. The present study sought to overcome some of these limitations for the first time by assessing the individual toxicological effects of aerosols generated from e-liquid base components using a 4th generation Pod-Mod device.

PG and VG are humectants used as universal solvents in all e-liquids and account for at least 90% of the total composition of e-liquids [45]. While PG and VG are classified GRAS by the FDA following oral ingestion studies, concerns have been raised about their pulmonary innocuity. Indeed, PG and VG have been shown to generate numerous carbonyls thermal degradation products, primarily formaldehyde and its derivatives, all of which are harmful to human health [46]. Interestingly, the concentration and number of carbonyls present in the aerosol of EC has been found to be closely correlated with the heating temperature of the coil, which is partially related to the operating settings of the device, such as atomizer resistance, voltage, and power [47, 48]. Although 4th generation devices operate at a lower temperature (≈ 200 °C) than 3rd generation devices, carbonyl compounds have been detected in the aerosol of JUUL device at concentrations exceeding the acute minimum risk level of public health agencies [49]. In the present study, humectants were found to be cytotoxic from about 330 µg/cm² (20 puffs) and at a 70/30 PG/VG ratio, whereas lower doses and both the 50/50 and 30/70 ratios had no effect. It has been shown that aerosolisation of pure PG from a low-power 2nd generation device (2.4 Ω atomizer, 3.4 V voltage, and 9.6 W output power) resulted in significantly higher carbonyl levels than pure VG, suggesting that concentration of PG was the main source of harmful thermal degradation products [50]. Since JUUL device operate at similar settings (1.6 Ω, 3.8 V, and 8.1 W) [28], the cytotoxicity of humectants observed in our work may be related to a higher carbonyl generation in 70/30 PG/VG aerosols than in 50/50 and 30/70 PG/VG aerosols.

The cytotoxicity of PG/VG at the air-liquid interface on respiratory cells has been investigated in a limited number of in vitro studies with mixed outcomes. Using a 2nd generation EC with JUUL-like electrical operating settings (2.8 Ω, 3.6 V, and 4.63 W), Antherieu et al. found no cytotoxicity of a 65/35 PG/VG aerosol in BEAS-2B cells up to 500 puffs [51]. However, aerosols and potentially related toxicants were air-diluted prior to exposure, which may underestimate their cytotoxicity [52]. Indeed, undiluted 70/30 PG/VG aerosols significantly decreased cell viability in Calu-3 cells above a threshold of 15 puffs [53], while 20 undiluted puffs of pure PG, but not 100% VG aerosols increased LDH release in 16HBE cells [54]. It should be noted that both studies used high-power devices (40–85 W) that can deliver extremely high doses (up to 1500 µg/cm²) and potentially increased amounts of thermal degradation products. Although 70/30 PG/VG elicited a strong cytotoxic response, only a slight increase in the production of the proinflammatory cytokine IL-8 and the oxidative DNA damage biomarker 8-OHdG was observed. Consistent with our results, the production of IL -8 and glutathione levels were slightly increased in BEAS-2B cells exposed to a nicotine-free 65/35 PG/VG aerosol [51]. Furthermore, increasing the wattage from 40 to 85 W did not increase IL-8 production in cells exposed to either pure PG or a 55/45 PG/VG ratio [54], supporting the hypothesis that PG is not involved in a proinflammatory response. The same study, reported that 100% VG aerosol, but not pure PG aerosol, significantly increased the expression of several genes involved in oxidative stress leading to protein carbonylation, suggesting that VG could be a stronger mediator of oxidative stress than PG. This assumption is further supported by a higher generation of hydroxyl radicals in EC aerosol of pure VG than pure PG [55].

Nicotine is the main active ingredient in e-liquids and is a well-known inducer of adverse effects on various physiological systems, including the respiratory system [56]. We found that nicotine freebase aerosol did not exhibit intrinsic cytotoxicity and did not further increase the production of IL-8 compared with the nicotine-free 70/30 PG/VG aerosol. These results were also observed in another study in which no changes in BEAS-2B cell viability and release of IL-8 were observed after exposure to a 16 mg/mL nicotine aerosol in 65/35 PG/VG compared with solvents alone [51]. Similarly, exposure of NCI-H292 epithelial cells to increasing concentrations of freebase nicotine from 0 mg/mL to 24 mg/mL in pure PG did not result in significant differences in cell metabolic activity and viability as well as in IL -6 production [57], whereas IL-8 levels were unchanged in a primary 3D EpiAirway™ model exposed to a nicotine-free or 25 mg/mL freebase nicotine aerosol [58]. Although freebase nicotine was not involved in cytotoxicity and inflammation, our results showed a significant increase in 8-OHdG levels compared with the 70/30 PG/VG matrix. The ability of freebase nicotine to generate reactive oxygen species (ROS) is uncertain. In a cell-free assay, Caruso et al. reported that aerosolisation of a PG/VG formulation containing 15 mg/mL nicotine generated ROS in a dose-dependent manner, whereas a nicotine-free formulation did not [59]. Conversely, in an earlier study, nicotine was unlikely to be the sole contributor to increased ROS reactivity [60]. These discrepancies may explain the conflicting reports on the potential of freebase nicotine to induce oxidative stress. Indeed, glutathione levels were unchanged between cells exposed to aerosols of PG/VG alone or PG/VG with 16 mg/mL of freebase nicotine [51]. Similarly, aerosols of a commercial e-liquid containing 24 mg/mL of nicotine did not further increase the production of ROS compared to its nicotine-free counterpart, regardless of the cell type used [61]. These data contrast with a transcriptomic study revealing that NHBE cells exposed to nicotine-containing EC condensates showed greater expression of several genes related to oxidative stress response than nicotine-free condensates [62]. It is important to note that all of the above studies had significant differences in their experimental design, including cell types, e-cigarette devices, or exposure system, all of which could explain the variability regarding the oxidative potential of freebase nicotine. Nevertheless, our data suggest that freebase nicotine vaped from a 4th generation device can induce acute oxidative stress in respiratory epithelial cells. Although Pod-Mod devices have been initially designed to use nicotine salts, single use Pods can be easily refilled with other e-liquids including freebase nicotine [63].

The proliferation of 4th generation EC devices has led to a surge in the popularity of nicotine salts, which promise higher level of nicotine inhalation with less discomfort than freebase nicotine, an attractive feature for users seeking quick nicotine relieve [14, 64]. However, research on nicotine salts is still in its infancy, and there are limited data on their potential toxicity, especially when compared to freebase nicotine. We have shown that nicotine salts elicited different toxicological responses depending on the type of organic acid used. While nicotine salts did not further increase LDH release or decrease cell viability compared with the freebase form, both salts strongly induced IL-8 production, but only nicotine benzoate increased 8-OHdG levels. Similarly, Escobar et al. found no differences in released LDH in primary human nasal cells exposed to nicotine salts or freebase aerosols from a 3rd generation device [34]. However, the protonation state of nicotine strongly influenced the amount and type of cytokines secreted. Specifically, both forms of nicotine were found to significantly increase IL-8 levels compared to air-exposed cells, but nicotine salts only slightly increased IL-8 production compared to freebase. This could be due to the presence of lactic acid in the tested e-liquids, a compound produced naturally by the body as a metabolic substrate, whereas the benzoic and salicylic acid used in our study are exogenous compounds. Although benzoic acid and salicylic acid are GRAS as food preservatives or as exfoliants in cosmetics, their effects on the lungs are unknown and no data are currently available. Since we found that both organic acids synergicaly in combination with PG/VG solvents, they might also have contributed to the proinflammatory effects of nicotine salts. Furthermore, it cannot be excluded that the pattern of thermal degradation products of nicotine salicylate and benzoate may differ in terms of carbonyl and VOC composition, leading to different toxicological outcomes on the respiratory epithelium. However, this hypothesis should be further investigated by in-depth chemical characterization studies of the aerosols [65]. In addition to its proinflammatory effects, aerosol of nicotine benzoate resulted in higher levels of DNA oxidative stress 8-OHdG than freebase nicotine. These findings are supported by a recent study that reported higher expression of several oxidative stress-related genes and biomarkers in macrophages exposed to a flavoured nicotine benzoate salt-based e-liquid compared with its freebase counterpart [35]. However, few changes were observed in inflammation-related genes, suggesting that the toxicological effects of nicotine salts may be specific to both the cell type and organic acid. Finally, the lack of oxidative effects of nicotine salicylate compared with nicotine benzoate could be due to the known antioxidant properties of salicylic acid [66], but this assumption requires further investigations.

The mint flavour in the commercial salt-based e-liquids investigated in this study is one of the most popular [67]. Since we have previously shown that the PG/VG ratio is the main contributor to cytotoxicity, we compared the flavoured e-liquids to their corresponding PG/VG ratio (50/50 for Flavor Power Mint and 30/70 for JUUL Mint). Both commercial e-liquids significantly reduced cell viability and impaired cell membrane integrity, as indicated by the concomitant increase in released LDH. Thus, our data showed that aerosols of flavoured commercial salt-based e-liquids were cytotoxic regardless of the type of organic acid and the PG/VG ratio. However, it was not possible to conclusively determine that the flavours were the primary cause of cytotoxicity because unflavoured nicotine salts in 50/50 and 30/70 PG/VG ratios were not studied in this work. Although IL-8 and 8-OHdG levels were significantly increased in cells exposed to mint-flavoured e-liquids compared with cells exposed to air (data not shown), these effects could also not be attributed solely to the flavours alone because the proinflammatory or oxidative effects of the corresponding PG/VG ratios were not examined. Overall, our results are consistent with other studies reporting various toxicological effects of aerosols of flavoured nicotine salt-based e-liquids in macrophages [35] or in normal (BEAS-2B) and tumorigenic (H292) lung epithelial cell lines [68] using a 4th generation device. Strikingly, a recent study found that mice exposed to a JUUL Mint aerosol for 60 min daily for up to three months exhibited various inflammatory states in multiple organs including brain, lung, heart, and intestine [69]. Together with the present work, these data confirm that the complex chemical composition of commercial salt-based e-liquids can cause both in vitro acute and in vivo chronic harmful effects.

A key aspect when comparing our data with the literature is the effective dose applied to the cells. Overall, dosimetry data are often inadequate or poorly reported and there is considerable variability between studies. This variability in deposited dose is mostly explained by the diversity in exposure setups, ranging from homemade boxes to automated puffing machines [44]. Depending on the exposure setup, a fraction of aerosol could be lost in tubing. Aerosols may or may not also be diluted prior to cell exposure. Finally, aerosols can be applied to cells by two broadly different methods: gravitational settling and active negative pressure. We chose to expose the cells to undiluted aerosols, a recently highlighted method that facilitates comparisons of toxicological data on e-cigarettes between different exposure systems by reducing the variability introduced by the dilution step and avoiding dilution of potential toxicants [52]. Escobar et al. recently validated an exposure system based on undiluted EC aerosols [54]. Interestingly, the average deposited dose was approximately three times higher (1000 µg/cm²) than in our study (330 µg/cm²), a difference that could be related to a three times larger puff volume (166 mL vs. 55 mL). The dosimetry of EC aerosols raises the question of the clinical relevance of in vitro exposure. A relevant deposited dose has not yet been established and a recent clinical study showed that approximately 31–36% of a [^11^C]nicotine aerosol is deposited in the respiratory tract [70]. Furthermore, Azzopardi et al. predicted an in vivo aerosol dose of 104 to 260 µg/cm² based on 140 to 300 puffs per day and a total upper airway area of 2690 cm² [71]. This dose range is lower than that reported in this work (≈ 330 µg/cm²). Therefore, the dose of 20 puffs used in the present study may have overestimated the amount of aerosol that can actually be deposited in the lungs. Considering that ≈ 30% of the initial aerosol mass is deposited in the respiratory tract, only about 100 µg/cm² would have been deposited in our study, corresponding roughly to 5 puffs condition(≈ 82 µg/cm²), which was not found to be cytotoxic. However, this work was primarily aimed at investigating the relative acute cytotoxicity between aerosols from various e-liquids composition and further studies are needed to evaluate chronic toxicity of EC aerosols based on physiological low doses.

Our study has several strengths. First, aerosols were generated and applied to cells using a well-characterised and validated exposure system in terms of deposited dose, ensuring that cells were exposed to identical aerosol doses regardless of e-liquid composition. Second, we used a physiologically relevant air-liquid interface that allowed exposure to both the gas and particulate phases of the undiluted aerosol, thus avoiding the loss of potentially toxic substances due to their phase partitioning and volatility [72]. Third, the experimental design, based on e-liquids of controlled composition, the use of the same device and standardized exposure parameters, allowed relevant comparisons within the same study and provided valuable new insights into the intrinsic toxicity of the basic constituents of e-liquids. However, this work also has some limitations. Toxicological analyses were performed in cells exposed to only EC aerosols. Although it was found that all the major constituents of e-liquids are transferred to the aerosol [73], our data cannot be used to identify the compound directly involved in the cytotoxic response, as the toxicity of unvaporised e-liquids was not investigated. Therefore, further studies on the chemical characterization of the aerosol are needed to reveal possible correlations between the pattern of thermal degradation products and cellular toxicological responses. Another drawback could be the use of undiluted aerosols that do not reflect the actual dilution of the EC aerosol in the respiratory tract and thus represent a less realistic high dose exposure. However, the use of extreme conditions may be appropriate in acute toxicological studies by increasing the likelihood of eliciting a response to avoid overlooking small effects. Therefore, our data are representative of short-term exposure effects but should not be used to extrapolate potential long-term effects of chronic EC use. Finally, the cell model used in this study is an immortalized cancer cell line, which does not adequately recapitulate lung physiology and limits extrapolation of results to in vivo EC aerosol exposure. In addition, sensitivity to EC aerosol has been shown to vary by cell type, with immortalized cells being less sensitive than primary cells [74]. However, the use of a primary cell line can be costly, time consuming, and lead to increased variability. Therefore, the choice of an immortalized cell line was consistent with the experimental design of the study, which examined a wide range of e-liquid compositions that required high reproducibility.

## Conclusion

The chemical complexity of the thousands of e-liquids on the market and the proliferation of 4th generation devices reinforce the ongoing need for toxicological studies of EC aerosols. Our results suggest that the basic constituents present in almost all e-liquids can pilot specific toxicological responses. In ALI-exposed lung epithelial cells, the concentration of the e-liquid solvent PG was the main determinant of cytotoxicity, although it had a lesser effect on inflammation and oxidative stress. Organic acids alone did not induce further cytotoxicity compared with PG/VG solvents, but synergistically inflammation in presence of PG/VG. Our results also suggest that nicotine in its freebase form promotes oxidative stress, whereas its protonated form enhances inflammation regardless of the nature of organic acid. In addition, nicotine salts generated with benzoic acid appeared to be more harmful than salicylic acid-based nicotine salts by inducing higher oxidative stress levels. Although further studies are needed to thoroughly investigate the toxicity of aerosols from 4th generation devices, our data could be useful for future regulation of EC products by health authorities.

## Data Availability

The datasets used and/or analysed during the current study are available from the corresponding author on reasonable request.

## Abbreviations

8-OHdG: 8-hydroxy 2 deoxyguanosine
ALI: Air-liquid interface
EC: Electronic cigarette
FDA: Food and drug administration
GRAS: Generally regarded as safe
IL-8: Interleukine-8
LDH: Lactate dehydrogenase
PG: Propylene glycol
ROS: Reactive oxygen species
VG: Vegetable glycerine
VOC: Volatile organic compounds
